# Supplementary material for: Differential Transcript Profiles in Cumulus-Oocyte Complexes Originating from Pre-Ovulatory Follicles of Varied Physiological Maturity in Beef Cows
Source: Genes (Basel). 2021 Jun 10;12(6):893. doi: 10.3390/genes12060893 (PMC8227736; doi:10.3390/genes12060893)
Supplement: Supplementary file 1 [file genes-12-00893-s001.zip › Figure_S1.pdf]

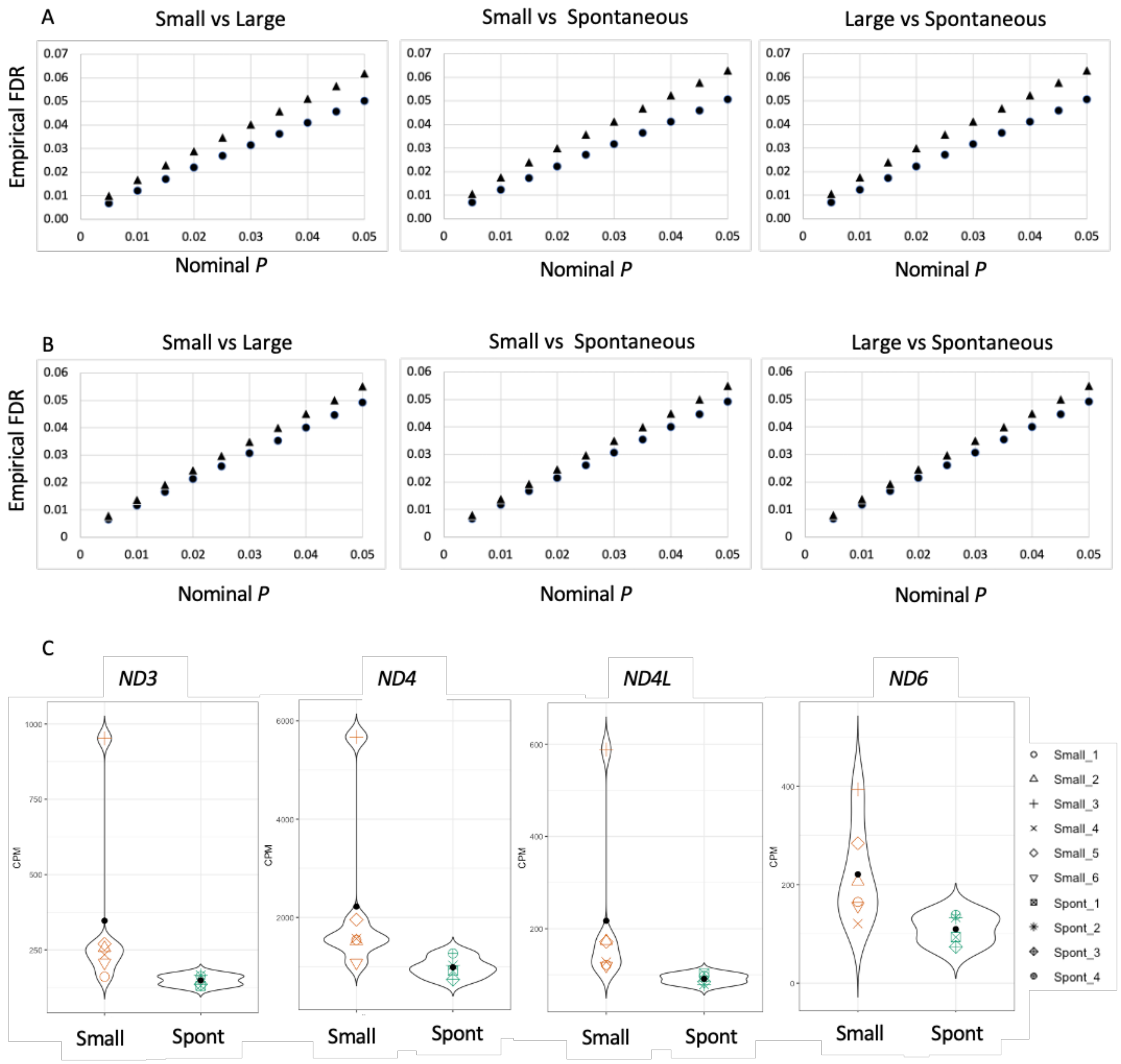

Figure S1. Plots relevant for DEG analysis. Relationship between nominal *P* values and empirical FDR calculated for edgeR (circle) and DESeq2 (triangle) analyses of differential transcript abundance in oocyte (Panel A) and CC pools (Panel B). Plots of counts per million in libraries of selected transcripts in the small versus spontaneous oocyte comparison. Plots highlight the rationale for validation removing library three from the analysis (Panel C).
