# Supplementary material for: Differential Transcript Profiles in Cumulus-Oocyte Complexes Originating from Pre-Ovulatory Follicles of Varied Physiological Maturity in Beef Cows
Source: Genes (Basel). 2021 Jun 10;12(6):893. doi: 10.3390/genes12060893 (PMC8227736; doi:10.3390/genes12060893)
Supplement: Supplementary file 1 [file genes-12-00893-s001.zip › File_S1.pdf]

Differential transcript profiles in cumulus-oocyte complexes originating from pre-ovulatory follicles of varied physiological maturity in beef cows.

Sarah E. Moorey [1*], Jenna M Monnig [2], Michael F. Smith [2], M. Sofia Ortega [2], Jonathon A. Green [2], Ky G. Pohler [3], G. Alan Bridges [4], Susanta K. Behura [2], and Thomas W. Geary [5]

[1]University of Tennessee, Knoxville, TN, USA, [2]University of Missouri, Columbia, MO, USA, [3]Texas A&M University, College Station, TX, USA, [4]University of Minnesota, St. Paul, MN, USA, [5]USDA-ARS Fort Keogh Livestock and Range Research Lab, Miles City, MT, USA
[*]Correspondence: smoorey5 at utk.edu

For questions, please contact Sarah Moorey (***smoorey5*** at ***utk.edu).***

Load the Required Libraries

```
library('edgeR')
library("DESeq2")
library("doParallel")
library ("bigmemory")
library("gtools")
library("permutations")
library("statmod")
```

Analysis of Cumulus Cells

Load and organize the count data

```
CC_counts=read.csv(file.choose())
CC <- CC_counts[,-1]
rownames(CC) <- CC_counts[,1]
```

Filter to remove lowly expressed transcripts

```
keep <- rowSums( cpm(CC) > 5 ) >= 5
CC<-CC[keep,]
```

DEG Small vs Large CC

edgeR

```
colnames(CC)
group<-factor(c("Small","Small", "Small","Small", "Small","Small", "Large", "Large","Large",
"Large", "Large", "Large", "Spont", "Spont","Spont", "Spont","Spont" ), levels=c("Small",
"Large", "Spont"))
design<-model.matrix(~group)
dds<-DGEList(count=CC, group=group)
dds<-estimateDisp(dds, design, robust=TRUE)
dds<-glmFit(dds, design)
dds<-glmLRT(dds,coef="groupLarge")
res_small_large_edgeR<-topTags(dds,n=Inf)$table
```

DESeq2

```
design<-model.matrix(~group)
colData<-data.frame("group"=group)
rownames(colData)<-colnames(CC)
dds<-DESeqDataSetFromMatrix(countData=CC, colData=colData, design= ~group)
dds<-DESeq(dds)
res_small_large_DeSeq<-results(dds, contrast=c("group","Small" ,"Large"),
pAdjustMethod="fdr", tidy=TRUE)
res_small_large_DeSeq<-res_small_large_DeSeq[with(res_small_large_DeSeq, order(pvalue)), ]
```

Merge results for edgeR and DESeq2

```
merged_res_small_large<-merge(res_small_large_edgeR, res_small_large_DeSeq,
by.x="row.names", by.y="row")
merged_res_small_large<-merged_res_small_large[merged_res_small_large$PValue<=0.01 &
merged_res_small_large$pvalue<=0.01,]
merged_res_small_large<-merged_res_small_large[with(merged_res_small_large,
order(PValue)),]
```

Calculate eFDR; this code should only be run on a multi-core server

Build permutation matrix

```
perm_without_replacement <- function(n, r){
        return(factorial(n)/factorial(n - r))
        perm_without_replacement(17,17)}

permutation_matrix<-matrix(,nrow=200000,ncol=17)

for (i in seq(1:200000)){
        sampling <- sample(1:17,17, replace = FALSE)
         if ( ! identical(sampling , c(1:17))){
        permutation_matrix[i,]<-sample(1:17,17, replace = FALSE)  }}

permutation_matrix<-permutation_matrix[!duplicated(permutation_matrix),]
permutation_matrix<-permutation_matrix[sample(1:200000 , 10000),]

rand<-dim(permutation_matrix)[1]

sequence.pvalue<-seq(0.005, 0.05, 0.005)

results <- filebacked.big.matrix(length(sequence.pvalue),rand, type="double", init=0,
separated=FALSE, backingfile="incidence_matrix.bin", descriptor="incidence_matrix.desc")
mdesc_result<- describe(results)
```

Calculate eFDR for edgeR

```
no_cores <- detectCores() - 1
cl <- makeCluster(no_cores)
registerDoParallel(cl)

results[,]<-foreach(i = sequence.pvalue, .combine='rbind', .inorder=TRUE,
.packages=c("edgeR","bigmemory"), .verbose=TRUE) %:%

  foreach(j = 1:rand, .combine='cbind', .inorder=FALSE,.packages=c("edgeR","bigmemory"),
.verbose=TRUE ) %dopar% {
        group<-c("Small","Small","Small","Small","Small",
        "Small","Large","Large","Large","Large", "Large", "Large", "Spont", "Spont", "Spont",
        "Spont", "Spont")
        group<-group[permutation_matrix[j,]]
        group<-factor(group, levels=c("Small","Large", "Spont"))
        design <- model.matrix(~group)
        dds<-DGEList(count=CC, group=group)
        dds<-estimateDisp(dds, design, robust=TRUE)
        dds <- glmFit(dds, design)
        dds<-glmLRT(dds,coef="groupLarge")
         length(which(topTags(dds,n=Inf)$table$PValue <= i)) }

stopCluster(cl)
total.rand <- rand * dim(CC)[1]
qvalue<-data.frame(raw.pvalue = sequence.pvalue,
           e.pvalue.edgeR= (rowSums(results[,]+1))/(total.rand+1),
           e.pvalue.edgeR.round= round((rowSums(results[,]+1))/(total.rand+1) ,4))

rm(results)
system("rm incidence_matrix.bin")
system("rm incidence_matrix.desc")
```

Calculate eFDR for DESeq2

```
results.rand <- filebacked.big.matrix(length(sequence.pvalue),rand, type="double", init=0,
separated=FALSE,  backingfile="incidence_matrix.bin", descriptor="incidence_matrix.desc")
mdesc_result<- describe(results.rand)

no_cores <- detectCores() - 1
cl <- makeCluster(no_cores)
registerDoParallel(cl)

results.rand[,]<-foreach(i = sequence.pvalue, .combine='rbind', .inorder=TRUE,
.packages=c("DESeq2","bigmemory"), .verbose=TRUE) %:%
```

```
foreach(j = 1:rand, .combine='cbind', .inorder=FALSE,.packages=c("DESeq2","bigmemory"),
.verbose=TRUE ) %dopar% {
        group<c("Small","Small","Small","Small","Small","Small","Large","Large","Large","Large"
        ,"Large","Large", "Spont", "Spont", "Spont", "Spont", "Spont")
        group<-group[permutation_matrix[j,]]
        group<-factor(group, levels=c("Small","Large", "Spont"))
        design <- model.matrix(~group)
        colData<-data.frame("group"=group)
        rownames(colData)<-colnames(CC)
        dds<-DESeqDataSetFromMatrix(countData=CC, colData=colData, design= ~group)
        dds <- DESeq(dds)
        dds<- results(dds, contrast=c("group","Small" ,"Large"), pAdjustMethod="fdr",
        tidy=TRUE)
        length(which(dds$pvalue <= i))  }

stopCluster(cl)

results.rand[1:5,1:5]
total.rand <- rand * dim(CC)[1]

qvalue2<-data.frame(raw.pvalue = sequence.pvalue,
          e.pvalue.DESEQ2= (rowSums(results.rand[,]+1))/(total.rand+1),
          e.pvalue.DESEQ2.round= round((rowSums(results.rand[,]+1))/(total.rand+1) ,4))

qvalue3<-cbind(qvalue,qvalue2)

rm(results.rand)
system("rm incidence_matrix.bin")
system("rm incidence_matrix.desc")
```

DEG Small vs Spontaneous CC

---

edgeR

```
group<-factor(c("Small","Small", "Small","Small", "Small","Small", "Large", "Large","Large",
"Large", "Large", "Large", "Spont", "Spont","Spont", "Spont","Spont" ), levels=c("Small",
"Large", "Spont"))
design<-model.matrix(~group)
dds<-DGEList(count=CC, group=group)
dds<-estimateDisp(dds, design, robust=TRUE)
dds<-glmFit(dds, design)
dds<-glmLRT(dds,coef="groupSpont")
res_small_spont_edgeR<-topTags(dds,n=Inf)$table
```

DESeq2

```
design<-model.matrix(~group)
```

```
colData<-data.frame("group"=group)
rownames(colData)<-colnames(CC)
dds<-DESeqDataSetFromMatrix(countData=CC, colData=colData, design= ~group)
dds<-DESeq(dds)
res_small_spont_DeSeq<-results(dds, contrast=c("group","Small" ,"Spont"),
pAdjustMethod="fdr", tidy=TRUE)
res_small_spont_DeSeq<-res_small_spont_DeSeq[with(res_small_spont_DeSeq,
order(pvalue)), ]
```

Merge results for edgeR and DESeq2

```
merged_res_small_spont<-merge(res_small_spont_edgeR, res_small_spont_DeSeq,
by.x="row.names", by.y="row")
merged_res_small_spont<-merged_res_small_spont[merged_res_small_spont$PValue<=0.01
& merged_res_small_spont$pvalue<=0.01,]
merged_res_small_spont<-merged_res_small_spont[with(merged_res_small_spont,
order(PValue)), ]
```

Calculate eFDR; this code should only be run on a multi-core server

Build permutation matrix

```
perm_without_replacement <- function(n, r){
	return(factorial(n)/factorial(n - r))
	perm_without_replacement(17,17)}

permutation_matrix<-matrix(,nrow=200000,ncol=17)

for (i in seq(1:200000)){
	sampling <- sample(1:17,17, replace = FALSE)
	 if ( ! identical(sampling , c(1:17))){
	permutation_matrix[i,]<-sample(1:17,17, replace = FALSE)  }}

permutation_matrix<-permutation_matrix[!duplicated(permutation_matrix),]
permutation_matrix<-permutation_matrix[sample(1:200000 , 10000),]

rand<-dim(permutation_matrix)[1]

sequence.pvalue<-seq(0.005, 0.05, 0.005)

results <- filebacked.big.matrix(length(sequence.pvalue),rand, type="double", init=0,
separated=FALSE, backingfile="incidence_matrix.bin", descriptor="incidence_matrix.desc")
mdesc_result<- describe(results)
```

Calculate eFDR for edgeR

```
no_cores <- detectCores() - 1
```

```
cl <- makeCluster(no_cores)
registerDoParallel(cl)

results[,]<-foreach(i = sequence.pvalue, .combine='rbind', .inorder=TRUE,
.packages=c("edgeR","bigmemory"), .verbose=TRUE) %:%

 foreach(j = 1:rand, .combine='cbind', .inorder=FALSE,.packages=c("edgeR","bigmemory"),
.verbose=TRUE ) %dopar% {
	group<-c("Small","Small","Small","Small","Small",
	"Small","Large","Large","Large","Large", "Large", "Large", "Spont", "Spont", "Spont",
	"Spont", "Spont")
	group<-group[permutation_matrix[j,]]
	group<-factor(group, levels=c("Small","Large", "Spont"))
	design <- model.matrix(~group)
	dds<-DGEList(count=CC, group=group)
	dds<-estimateDisp(dds, design, robust=TRUE)
	dds <- glmFit(dds, design)
	dds<-glmLRT(dds,coef="groupSpont")
	 length(which(topTags(dds,n=Inf)$table$PValue <= i)) }

stopCluster(cl)
total.rand <- rand * dim(CC)[1]
qvalue<-data.frame(raw.pvalue = sequence.pvalue,
		e.pvalue.edgeR= (rowSums(results[,]+1))/(total.rand+1),
		e.pvalue.edgeR.round= round((rowSums(results[,]+1))/(total.rand+1) ,4))

rm(results)
system("rm incidence_matrix.bin")
system("rm incidence_matrix.desc")
```

Calculate eFDR for DESeq2

```
results.rand <- filebacked.big.matrix(length(sequence.pvalue),rand, type="double", init=0,
separated=FALSE,  backingfile="incidence_matrix.bin", descriptor="incidence_matrix.desc")
mdesc_result<- describe(results.rand)

no_cores <- detectCores() - 1
cl <- makeCluster(no_cores)
registerDoParallel(cl)

results.rand[,]<-foreach(i = sequence.pvalue, .combine='rbind', .inorder=TRUE,
.packages=c("DESeq2","bigmemory"), .verbose=TRUE) %:%

foreach(j = 1:rand, .combine='cbind', .inorder=FALSE,.packages=c("DESeq2","bigmemory"),
.verbose=TRUE ) %dopar% {
```

```
        group<c("Small","Small","Small","Small","Small","Small","Large","Large","Large","Large"
        ,"Large","Large", "Spont", "Spont", "Spont", "Spont", "Spont")
        group<-group[permutation_matrix[j,]]
        group<-factor(group, levels=c("Small","Large", "Spont"))
        design <- model.matrix(~group)
        colData<-data.frame("group"=group)
        rownames(colData)<-colnames(CC)
        dds<-DESeqDataSetFromMatrix(countData=CC, colData=colData, design= ~group)
        dds <- DESeq(dds)
        dds<- results(dds, contrast=c("group","Small" ,"Spont"), pAdjustMethod="fdr",
        tidy=TRUE)
        length(which(dds$pvalue <= i))  }

stopCluster(cl)

results.rand[1:5,1:5]
total.rand <- rand * dim(CC)[1]

qvalue2<-data.frame(raw.pvalue = sequence.pvalue,
        e.pvalue.DESEQ2= (rowSums(results.rand[,]+1))/(total.rand+1),
        e.pvalue.DESEQ2.round= round((rowSums(results.rand[,]+1))/(total.rand+1) ,4))

qvalue3<-cbind(qvalue,qvalue2)

rm(results.rand)
system("rm incidence_matrix.bin")
system("rm incidence_matrix.desc")
```

DEG Large vs Spontaneous CC

edgeR

```
group<-factor(c("Small","Small", "Small","Small", "Small","Small", "Large", "Large","Large",
"Large", "Large", "Large", "Spont", "Spont","Spont", "Spont","Spont" ), levels=c("Large",
"Small", "Spont"))
design<-model.matrix(~group)
dds<-DGEList(count=CC, group=group)
dds<-estimateDisp(dds, design, robust=TRUE)
dds<-glmFit(dds, design)
dds<-glmLRT(dds,coef="groupSpont")
res_large_spont_edgeR<-topTags(dds,n=Inf)$table
```

DESeq2

```
design<-model.matrix(~group)
colData<-data.frame("group"=group)
```

```
rownames(colData)<-colnames(CC)
dds<-DESeqDataSetFromMatrix(countData=CC, colData=colData, design= ~group)
dds<-DESeq(dds)
res_large_spont_DeSeq<-results(dds, contrast=c("group","Large" ,"Spont"),
pAdjustMethod="fdr", tidy=TRUE)
res_large_spont_DeSeq<-res_large_spont_DeSeq[with(res_large_spont_DeSeq, order(pvalue)),
]
```

Merge results for edgeR and DESeq2

```
merged_res_large_spont<-merge(res_large_spont_edgeR, res_large_spont_DeSeq,
by.x="row.names", by.y="row")
merged_res_large_spont<-merged_res_large_spont[merged_res_large_spont$PValue<=0.01 &
merged_res_large_spont$pvalue<=0.01,]
merged_res_large_spont<-merged_res_large_spont[with(merged_res_large_spont,
order(PValue)), ]
```

Calculate eFDR; this code should only be run on a multi-core server

Build permutation matrix

```
perm_without_replacement <- function(n, r){
        return(factorial(n)/factorial(n - r))
        perm_without_replacement(17,17)}

permutation_matrix<-matrix(,nrow=200000,ncol=17)

for (i in seq(1:200000)){
        sampling <- sample(1:17,17, replace = FALSE)
         if ( ! identical(sampling , c(1:17))){
        permutation_matrix[i,]<-sample(1:17,17, replace = FALSE)  }}

permutation_matrix<-permutation_matrix[!duplicated(permutation_matrix),]
permutation_matrix<-permutation_matrix[sample(1:200000 , 10000),]

rand<-dim(permutation_matrix)[1]

sequence.pvalue<-seq(0.005, 0.05, 0.005)

results <- filebacked.big.matrix(length(sequence.pvalue),rand, type="double", init=0,
separated=FALSE, backingfile="incidence_matrix.bin", descriptor="incidence_matrix.desc")
mdesc_result<- describe(results)
```

Calculate eFDR for edgeR

```
no_cores <- detectCores() - 1
cl <- makeCluster(no_cores)
```

```
registerDoParallel(cl)

results[,]<-foreach(i = sequence.pvalue, .combine='rbind', .inorder=TRUE,
.packages=c("edgeR","bigmemory"), .verbose=TRUE) %:%

  foreach(j = 1:rand, .combine='cbind', .inorder=FALSE,.packages=c("edgeR","bigmemory"),
.verbose=TRUE ) %dopar% {
        group<-c("Small","Small","Small","Small","Small",
        "Small","Large","Large","Large","Large", "Large", "Large", "Spont", "Spont", "Spont",
        "Spont", "Spont")
          group<-group[permutation_matrix[j,]]
        group<-factor(group, levels=c("Large", “Small”, "Spont"))
        design <- model.matrix(~group)
        dds<-DGEList(count=CC, group=group)
        dds<-estimateDisp(dds, design, robust=TRUE)
        dds <- glmFit(dds, design)
        dds<-glmLRT(dds,coef="groupSpont")
         length(which(topTags(dds,n=Inf)$table$PValue <= i)) }

stopCluster(cl)
total.rand <- rand * dim(CC)[1]
qvalue<-data.frame(raw.pvalue = sequence.pvalue,
            e.pvalue.edgeR= (rowSums(results[,]+1))/(total.rand+1),
            e.pvalue.edgeR.round= round((rowSums(results[,]+1))/(total.rand+1) ,4))

rm(results)
system("rm incidence_matrix.bin")
system("rm incidence_matrix.desc")
```

Calculate eFDR for DESeq2

```
results.rand <- filebacked.big.matrix(length(sequence.pvalue),rand, type="double", init=0,
separated=FALSE,  backingfile="incidence_matrix.bin", descriptor="incidence_matrix.desc")
mdesc_result<- describe(results.rand)

no_cores <- detectCores() - 1
cl <- makeCluster(no_cores)
registerDoParallel(cl)

results.rand[,]<-foreach(i = sequence.pvalue, .combine='rbind', .inorder=TRUE,
.packages=c("DESeq2","bigmemory"), .verbose=TRUE) %:%

foreach(j = 1:rand, .combine='cbind', .inorder=FALSE,.packages=c("DESeq2","bigmemory"),
.verbose=TRUE ) %dopar% {
```

```
        group<c("Small","Small","Small","Small","Small","Small","Large","Large","Large","Large"
        ,"Large","Large", "Spont", "Spont", "Spont", "Spont", "Spont")
        group<-group[permutation_matrix[j,]]
        group<-factor(group, levels=c("Large", ”Small”, "Spont"))
        design <- model.matrix(~group)
        colData<-data.frame("group"=group)
        rownames(colData)<-colnames(CC)
        dds<-DESeqDataSetFromMatrix(countData=CC, colData=colData, design= ~group)
        dds <- DESeq(dds)
        dds<- results(dds, contrast=c("group","Large", “Spont”), pAdjustMethod="fdr",
        tidy=TRUE)
        length(which(dds$pvalue <= i))  }

stopCluster(cl)

results.rand[1:5,1:5]
total.rand <- rand * dim(CC)[1]

qvalue2<-data.frame(raw.pvalue = sequence.pvalue,
            e.pvalue.DESEQ2= (rowSums(results.rand[,]+1))/(total.rand+1),
            e.pvalue.DESEQ2.round= round((rowSums(results.rand[,]+1))/(total.rand+1) ,4))

qvalue3<-cbind(qvalue,qvalue2)

rm(results.rand)
system("rm incidence_matrix.bin")
system("rm incidence_matrix.desc")
```

Analysis of Oocytes

Load the oocyte count data

```
Oocyte_counts=read.csv(file.choose())
Oocyte <- Oocyte_counts[,-1]
rownames(Oocyte) <- Oocyte_counts[,1]
```

Filter to remove lowly expressed transcripts

```
keep <- rowSums( cpm(Oocyte) > 5 ) >= 4
Oocyte<-Oocyte[keep,]
```

DEG Small vs Large Oocytes

edgeR

```
colnames(Oocyte)
group<-factor(c("Small","Small", "Small","Small", "Small","Small", "Large", "Large","Large",
"Large", "Large", "Large", "Spont", "Spont","Spont", "Spont" ), levels=c("Small", "Large",
"Spont"))
design<-model.matrix(~group)
dds<-DGEList(count=Oocyte, group=group)
dds<-estimateDisp(dds, design, robust=TRUE)
dds<-glmFit(dds, design)
dds<-glmLRT(dds,coef="groupLarge")
res_small_large_edgeR<-topTags(dds,n=Inf)$table
```

DESeq2

```
design<-model.matrix(~group)
colData<-data.frame("group"=group)
rownames(colData)<-colnames(Oocyte)
dds<-DESeqDataSetFromMatrix(countData=Oocyte, colData=colData, design= ~group)
dds<-DESeq(dds)
res_small_large_DeSeq<-results(dds, contrast=c("group","Small" ,"Large"),
pAdjustMethod="fdr", tidy=TRUE)
res_small_large_DeSeq<-res_small_large_DeSeq[with(res_small_large_DeSeq, order(pvalue)), ]
```

Merge the results for edgeR and DESeq2

```
merged_res_small_large<-merge(res_small_large_edgeR, res_small_large_DeSeq,
by.x="row.names", by.y="row")
merged_res_small_large<-merged_res_small_large[merged_res_small_large$PValue<=0.01 &
merged_res_small_large$pvalue<=0.01,]
merged_res_small_large<-merged_res_small_large[with(merged_res_small_large,
order(PValue)), ]
```

Calculate eFDR; this code should only be run on a multi-core server

Build permutation matrix

```
perm_without_replacement <- function(n, r){
        return(factorial(n)/factorial(n - r))
        perm_without_replacement(16,16)}

permutation_matrix<-matrix(,nrow=200000,ncol=16)

for (i in seq(1:200000)){
        sampling <- sample(1:16,16, replace = FALSE)
         if ( ! identical(sampling , c(1:16))){
        permutation_matrix[i,]<-sample(1:16,16, replace = FALSE)  }}
```

```
permutation_matrix<-permutation_matrix[!duplicated(permutation_matrix),]
permutation_matrix<-permutation_matrix[sample(1:200000 , 10000),]

rand<-dim(permutation_matrix)[1]

sequence.pvalue<-seq(0.005, 0.05, 0.005)

results <- filebacked.big.matrix(length(sequence.pvalue),rand, type="double", init=0,
separated=FALSE, backingfile="incidence_matrix.bin", descriptor="incidence_matrix.desc")
mdesc_result<- describe(results)
```

Calculate eFDR for edgeR

```
no_cores <- detectCores() - 1
cl <- makeCluster(no_cores)
registerDoParallel(cl)

results[,]<-foreach(i = sequence.pvalue, .combine='rbind', .inorder=TRUE,
.packages=c("edgeR","bigmemory"), .verbose=TRUE) %:%

  foreach(j = 1:rand, .combine='cbind', .inorder=FALSE,.packages=c("edgeR","bigmemory"),
.verbose=TRUE ) %dopar% {
        group<-c("Small","Small","Small","Small","Small",
        "Small","Large","Large","Large","Large", "Large", "Large", "Spont", "Spont", "Spont",
        "Spont")
        group<-group[permutation_matrix[j,]]
        group<-factor(group, levels=c(“Small”, “Large”, "Spont"))
        design <- model.matrix(~group)
        dds<-DGEList(count=CC, group=group)
        dds<-estimateDisp(dds, design, robust=TRUE)
        dds <- glmFit(dds, design)
        dds<-glmLRT(dds,coef="groupLarge")
         length(which(topTags(dds,n=Inf)$table$PValue <= i)) }

stopCluster(cl)
total.rand <- rand * dim(Oocyte)[1]
qvalue<-data.frame(raw.pvalue = sequence.pvalue,
            e.pvalue.edgeR= (rowSums(results[,]+1))/(total.rand+1),
            e.pvalue.edgeR.round= round((rowSums(results[,]+1))/(total.rand+1) ,4))

rm(results)
system("rm incidence_matrix.bin")
system("rm incidence_matrix.desc")
```

Calculate eFDR for DESeq2

```
results.rand <- filebacked.big.matrix(length(sequence.pvalue),rand, type="double", init=0,
separated=FALSE,  backingfile="incidence_matrix.bin", descriptor="incidence_matrix.desc")
mdesc_result<- describe(results.rand)

no_cores <- detectCores() - 1
cl <- makeCluster(no_cores)
registerDoParallel(cl)

results.rand[,]<-foreach(i = sequence.pvalue, .combine='rbind', .inorder=TRUE,
.packages=c("DESeq2","bigmemory"), .verbose=TRUE) %:%

foreach(j = 1:rand, .combine='cbind', .inorder=FALSE,.packages=c("DESeq2","bigmemory"),
.verbose=TRUE ) %dopar% {
        group<c("Small","Small","Small","Small","Small","Small","Large","Large","Large","Large"
        ,"Large","Large", "Spont", "Spont", "Spont", "Spont")
        group<-group[permutation_matrix[j,]]
        group<-factor(group, levels=c(”Small”, “Large”, "Spont"))
        design <- model.matrix(~group)
        colData<-data.frame("group"=group)
        rownames(colData)<-colnames(CC)
        dds<-DESeqDataSetFromMatrix(countData=CC, colData=colData, design= ~group)
        dds <- DESeq(dds)
        dds<- results(dds, contrast=c("group", “Small”, "Large"), pAdjustMethod="fdr",
        tidy=TRUE)
        length(which(dds$pvalue <= i))  }

stopCluster(cl)

results.rand[1:5,1:5]
total.rand <- rand * dim(Oocyte)[1]

qvalue2<-data.frame(raw.pvalue = sequence.pvalue,
            e.pvalue.DESEQ2= (rowSums(results.rand[,]+1))/(total.rand+1),
            e.pvalue.DESEQ2.round= round((rowSums(results.rand[,]+1))/(total.rand+1) ,4))

qvalue3<-cbind(qvalue,qvalue2)

rm(results.rand)
system("rm incidence_matrix.bin")
system("rm incidence_matrix.desc")
```

DEG Small vs Spont Oocytes

edgeR

```
group<-factor(c("Small","Small", "Small","Small", "Small","Small", "Large", "Large","Large",
"Large", "Large", "Large", "Spont", "Spont","Spont", "Spont" ), levels=c("Small", "Large",
"Spont"))
design<-model.matrix(~group)
dds<-DGEList(count=Oocyte, group=group)
dds<-estimateDisp(dds, design, robust=TRUE)
dds<-glmFit(dds, design)
dds<-glmLRT(dds,coef="groupSpont")
res_small_spont_edgeR<-topTags(dds,n=Inf)$table
head(res_small_spont_edgeR,n=30)
```

DESeq2

```
design<-model.matrix(~group)
colData<-data.frame("group"=group)
rownames(colData)<-colnames(Oocyte)
dds<-DESeqDataSetFromMatrix(countData=Oocyte, colData=colData, design= ~group)
dds<-DESeq(dds)
res_small_spont_DeSeq<-results(dds, contrast=c("group","Small" ,"Spont"),
pAdjustMethod="fdr", tidy=TRUE)
res_small_spont_DeSeq<-res_small_spont_DeSeq[with(res_small_spont_DeSeq,
order(pvalue)), ]
head(res_small_spont_DeSeq,n=30)
```

Merge the results for edgeR and DESeq2

```
merged_res_small_spont<-merge(res_small_spont_edgeR, res_small_spont_DeSeq,
by.x="row.names", by.y="row")
merged_res_small_spont<-merged_res_small_spont[merged_res_small_spont$PValue<=0.01
& merged_res_small_spont$pvalue<=0.01,]
dim(merged_res_small_spont) #128
head(merged_res_small_spont)
merged_res_small_spont<-merged_res_small_spont[with(merged_res_small_spont,
order(PValue)), ]
```

Calculate eFDR; this code should only be run on a multi-core server

Build permutation matrix

```
perm_without_replacement <- function(n, r){
        return(factorial(n)/factorial(n - r))
        perm_without_replacement(16,16)}

permutation_matrix<-matrix(,nrow=200000,ncol=16)
```

```
for (i in seq(1:200000)){
	sampling <- sample(1:16,16, replace = FALSE)
	 if ( ! identical(sampling , c(1:16))){
	permutation_matrix[i,]<-sample(1:16,16, replace = FALSE)  }}

permutation_matrix<-permutation_matrix[!duplicated(permutation_matrix),]
permutation_matrix<-permutation_matrix[sample(1:200000 , 10000),]

rand<-dim(permutation_matrix)[1]

sequence.pvalue<-seq(0.005, 0.05, 0.005)

results <- filebacked.big.matrix(length(sequence.pvalue),rand, type="double", init=0,
separated=FALSE, backingfile="incidence_matrix.bin", descriptor="incidence_matrix.desc")
mdesc_result<- describe(results)
```

Calculate eFDR for edgeR

```
no_cores <- detectCores() - 1
cl <- makeCluster(no_cores)
registerDoParallel(cl)

results[,]<-foreach(i = sequence.pvalue, .combine='rbind', .inorder=TRUE,
.packages=c("edgeR","bigmemory"), .verbose=TRUE) %:%

  foreach(j = 1:rand, .combine='cbind', .inorder=FALSE,.packages=c("edgeR","bigmemory"),
.verbose=TRUE ) %dopar% {
	group<-c("Small","Small","Small","Small","Small",
	"Small","Large","Large","Large","Large", "Large", "Large", "Spont", "Spont", "Spont",
	"Spont")
	group<-group[permutation_matrix[j,]]
	group<-factor(group, levels=c(“Small”, “Large”, "Spont"))
	design <- model.matrix(~group)
	dds<-DGEList(count=CC, group=group)
	dds<-estimateDisp(dds, design, robust=TRUE)
	dds <- glmFit(dds, design)
	dds<-glmLRT(dds,coef="groupSpont")
	 length(which(topTags(dds,n=Inf)$table$PValue <= i)) }

stopCluster(cl)
total.rand <- rand * dim(Oocyte)[1]
qvalue<-data.frame(raw.pvalue = sequence.pvalue,
		e.pvalue.edgeR= (rowSums(results[,]+1))/(total.rand+1),
		e.pvalue.edgeR.round= round((rowSums(results[,]+1))/(total.rand+1) ,4))
```

```
rm(results)
system("rm incidence_matrix.bin")
system("rm incidence_matrix.desc")
```

Calculate eFDR for DESeq2

```
results.rand <- filebacked.big.matrix(length(sequence.pvalue),rand, type="double", init=0,
separated=FALSE,  backingfile="incidence_matrix.bin", descriptor="incidence_matrix.desc")
mdesc_result<- describe(results.rand)

no_cores <- detectCores() - 1
cl <- makeCluster(no_cores)
registerDoParallel(cl)

results.rand[,]<-foreach(i = sequence.pvalue, .combine='rbind', .inorder=TRUE,
.packages=c("DESeq2","bigmemory"), .verbose=TRUE) %:%

foreach(j = 1:rand, .combine='cbind', .inorder=FALSE,.packages=c("DESeq2","bigmemory"),
.verbose=TRUE ) %dopar% {
        group<c("Small","Small","Small","Small","Small","Small","Large","Large","Large","Large"
        ,"Large","Large", "Spont", "Spont", "Spont", "Spont")
        group<-group[permutation_matrix[j,]]
        group<-factor(group, levels=c( ”Small”, “Large”, "Spont"))
        design <- model.matrix(~group)
        colData<-data.frame("group"=group)
        rownames(colData)<-colnames(CC)
        dds<-DESeqDataSetFromMatrix(countData=CC, colData=colData, design= ~group)
        dds <- DESeq(dds)
        dds<- results(dds, contrast=c("group","Small", “Spont”), pAdjustMethod="fdr",
        tidy=TRUE)
        length(which(dds$pvalue <= i))  }

stopCluster(cl)

results.rand[1:5,1:5]
total.rand <- rand * dim(Oocyte)[1]

qvalue2<-data.frame(raw.pvalue = sequence.pvalue,
            e.pvalue.DESEQ2= (rowSums(results.rand[,]+1))/(total.rand+1),
            e.pvalue.DESEQ2.round= round((rowSums(results.rand[,]+1))/(total.rand+1) ,4))

qvalue3<-cbind(qvalue,qvalue2)

rm(results.rand)
system("rm incidence_matrix.bin")
```

```
system("rm incidence_matrix.desc")
system("rm incidence_matrix.desc")
```

DEG Large vs Spont Oocyte

---

edgeR

```
group<-factor(c("Small","Small", "Small","Small", "Small","Small", "Large", "Large","Large",
"Large", "Large", "Large", "Spont", "Spont", "Spont","Spont" ), levels=c("Large", "Small",
"Spont"))
design<-model.matrix(~group)
dds<-DGEList(count=Oocyte, group=group)
dds<-estimateDisp(dds, design, robust=TRUE)
dds<-glmFit(dds, design)
dds<-glmLRT(dds,coef="groupSpont")
res_large_spont_edgeR<-topTags(dds,n=Inf)$table
head(res_large_spont_edgeR,n=30)
```

DESeq2

```
design<-model.matrix(~group)
colData<-data.frame("group"=group)
rownames(colData)<-colnames(Oocyte)
dds<-DESeqDataSetFromMatrix(countData=Oocyte, colData=colData, design= ~group)
dds<-DESeq(dds)
res_large_spont_DeSeq<-results(dds, contrast=c("group","Large" ,"Spont"),
pAdjustMethod="fdr", tidy=TRUE)
res_large_spont_DeSeq<-res_large_spont_DeSeq[with(res_large_spont_DeSeq, order(pvalue)),
]
head(res_large_spont_DeSeq,n=30)
```

Merge the results for edgeR and DESeq2

```
merged_res_large_spont<-merge(res_large_spont_edgeR, res_large_spont_DeSeq,
by.x="row.names", by.y="row")
merged_res_large_spont<-merged_res_large_spont[merged_res_large_spont$PValue<=0.01 &
merged_res_large_spont$pvalue<=0.01,]
head(merged_res_large_spont)
merged_res_large_spont<-merged_res_large_spont[with(merged_res_large_spont,
order(PValue)), ]
```

Calculate eFDR; this code should only be run on a multi-core server

Build permutation matrix

```
perm_without_replacement <- function(n, r){
        return(factorial(n)/factorial(n - r))
        perm_without_replacement(16,16)}
```

```
permutation_matrix<-matrix(,nrow=200000,ncol=16)

for (i in seq(1:200000)){
        sampling <- sample(1:16,16, replace = FALSE)
         if ( ! identical(sampling , c(1:16))){
        permutation_matrix[i,]<-sample(1:16,16, replace = FALSE)  }}

permutation_matrix<-permutation_matrix[!duplicated(permutation_matrix),]
permutation_matrix<-permutation_matrix[sample(1:200000 , 10000),]

rand<-dim(permutation_matrix)[1]

sequence.pvalue<-seq(0.005, 0.05, 0.005)

results <- filebacked.big.matrix(length(sequence.pvalue),rand, type="double", init=0,
separated=FALSE, backingfile="incidence_matrix.bin", descriptor="incidence_matrix.desc")
mdesc_result<- describe(results)
```

Calculate eFDR for edgeR

```
no_cores <- detectCores() - 1
cl <- makeCluster(no_cores)
registerDoParallel(cl)

results[,]<-foreach(i = sequence.pvalue, .combine='rbind', .inorder=TRUE,
.packages=c("edgeR","bigmemory"), .verbose=TRUE) %:%

  foreach(j = 1:rand, .combine='cbind', .inorder=FALSE,.packages=c("edgeR","bigmemory"),
.verbose=TRUE ) %dopar% {
        group<-c("Small","Small","Small","Small","Small",
        "Small","Large","Large","Large","Large", "Large", "Large", "Spont", "Spont", "Spont",
        "Spont")
        group<-group[permutation_matrix[j,]]
        group<-factor(group, levels=c("Large", “Small”, "Spont"))
        design <- model.matrix(~group)
        dds<-DGEList(count=CC, group=group)
        dds<-estimateDisp(dds, design, robust=TRUE)
        dds <- glmFit(dds, design)
        dds<-glmLRT(dds,coef="groupSpont")
         length(which(topTags(dds,n=Inf)$table$PValue <= i)) }

stopCluster(cl)
total.rand <- rand * dim(Oocyte)[1]
qvalue<-data.frame(raw.pvalue = sequence.pvalue,
```

```
        e.pvalue.edgeR= (rowSums(results[,]+1))/(total.rand+1),
        e.pvalue.edgeR.round= round((rowSums(results[,]+1))/(total.rand+1) ,4))

rm(results)
system("rm incidence_matrix.bin")
system("rm incidence_matrix.desc")
```

Calculate eFDR for DESeq2

```
results.rand <- filebacked.big.matrix(length(sequence.pvalue),rand, type="double", init=0,
separated=FALSE,  backingfile="incidence_matrix.bin", descriptor="incidence_matrix.desc")
mdesc_result<- describe(results.rand)

no_cores <- detectCores() - 1
cl <- makeCluster(no_cores)
registerDoParallel(cl)

results.rand[,]<-foreach(i = sequence.pvalue, .combine='rbind', .inorder=TRUE,
.packages=c("DESeq2","bigmemory"), .verbose=TRUE) %:%

foreach(j = 1:rand, .combine='cbind', .inorder=FALSE,.packages=c("DESeq2","bigmemory"),
.verbose=TRUE ) %dopar% {
    group<c("Small","Small","Small","Small","Small","Small","Large","Large","Large","Large"
    ,"Large","Large", "Spont", "Spont", "Spont", "Spont")
    group<-group[permutation_matrix[j,]]
    group<-factor(group, levels=c("Large", ”Small”, "Spont"))
    design <- model.matrix(~group)
    colData<-data.frame("group"=group)
    rownames(colData)<-colnames(CC)
    dds<-DESeqDataSetFromMatrix(countData=CC, colData=colData, design= ~group)
    dds <- DESeq(dds)
    dds<- results(dds, contrast=c("group", “Large”, “Spont”), pAdjustMethod="fdr",
    tidy=TRUE)
    length(which(dds$pvalue <= i))  }

stopCluster(cl)

results.rand[1:5,1:5]
total.rand <- rand * dim(Oocyte)[1]

qvalue2<-data.frame(raw.pvalue = sequence.pvalue,
        e.pvalue.DESEQ2= (rowSums(results.rand[,]+1))/(total.rand+1),
        e.pvalue.DESEQ2.round= round((rowSums(results.rand[,]+1))/(total.rand+1) ,4))

qvalue3<-cbind(qvalue,qvalue2)
```

```
rm(results.rand)
system("rm incidence_matrix.bin")
system("rm incidence_matrix.desc")
system("rm incidence_matrix.desc")
```

Analysis of Oocytes Minus Library Three

Re-load the oocyte count data

```
Oocyte_counts=read.csv(file.choose())
Oocyte <- Oocyte_counts[,-1]
rownames(Oocyte) <- Oocyte_counts[,1]
```

Remove library 3

```
Oocyte<-Oocyte[,-3]
head(Oocyte)
```

Filter to remove lowly expressed transcripts

```
keep <- rowSums( cpm(Oocyte) > 5 ) >= 4
Oocyte<-Oocyte[keep,]
head(Oocyte)
```

DEG Small vs Large Oocyte Minus Library Three

edgeR

```
group<-factor(c("Small","Small","Small", "Small","Small", "Large", "Large","Large", "Large", "Large",
"Large", "Large", "Spont", "Spont","Spont", "Spont" ), levels=c("Small", "Large", "Spont"))
design<-model.matrix(~group)
dds<-DGEList(count=Oocyte, group=group)
dds<-estimateDisp(dds, design, robust=TRUE)
dds<-glmFit(dds, design)
dds<-glmLRT(dds,coef="groupLarge")
res_small_large_edgeR<-topTags(dds,n=Inf)$table
head(res_small_large_edgeR,n=30)
```

DESeq2

```
design<-model.matrix(~group)
colData<-data.frame("group"=group)
rownames(colData)<-colnames(Oocyte)
dds<-DESeqDataSetFromMatrix(countData=Oocyte, colData=colData, design= ~group)
dds<-DESeq(dds)
res_small_large_DeSeq<-results(dds, contrast=c("group","Small" ,"Large"),
pAdjustMethod="fdr", tidy=TRUE)
res_small_large_DeSeq<-res_small_large_DeSeq[with(res_small_large_DeSeq, order(pvalue)), ]
```

```
head(res_small_large_DeSeq,n=30)
```

Merge the results for edgeR and DESeq2

```
merged_res_small_large_minus_3<-merge(res_small_large_edgeR, res_small_large_DeSeq,
by.x="row.names", by.y="row")
merged_res_small_large_minus_3<-
merged_res_small_large_minus_3[merged_res_small_large_minus_3$PValue<=0.01 &
merged_res_small_large_minus_3$pvalue<=0.01,]
dim(merged_res_small_large_minus_3)
head(merged_res_small_large_minus_3)
merged_res_small_large_minus_3<-
merged_res_small_large_minus_3[with(merged_res_small_large_minus_3, order(PValue)), ]
```

Merge the results for initial and validation analysis

```
merged_res_small_large_validated<-merge(merged_res_small_large,
merged_res_small_large_minus_3, by.x="row.names", by.y="row.names", all.x=TRUE,
all.y=FALSE)
```

DEG Small vs Spont Oocyte Minus Library Three

edgeR

```
group<-factor(c("Small", "Small","Small", "Small","Small", "Large", "Large","Large", "Large",
"Large", "Large", "Spont", "Spont","Spont", "Spont" ), levels=c("Small", "Large", "Spont"))
design<-model.matrix(~group)
dds<-DGEList(count=Oocyte, group=group)
dds<-estimateDisp(dds, design, robust=TRUE)
dds<-glmFit(dds, design)
dds<-glmLRT(dds,coef="groupSpont")
res_small_spont_edgeR<-topTags(dds,n=Inf)$table
head(res_small_spont_edgeR,n=30)
```

DESeq2

```
design<-model.matrix(~group)
colData<-data.frame("group"=group)
rownames(colData)<-colnames(Oocyte)
dds<-DESeqDataSetFromMatrix(countData=Oocyte, colData=colData, design= ~group)
dds<-DESeq(dds)
res_small_spont_DeSeq<-results(dds, contrast=c("group","Small" ,"Spont"),
pAdjustMethod="fdr", tidy=TRUE)
res_small_spont_DeSeq<-res_small_spont_DeSeq[with(res_small_spont_DeSeq,
order(pvalue)), ]
head(res_small_spont_DeSeq,n=30)
```

Merge the results for edgeR and DESeq2

```
merged_res_small_spont_minus_3<-merge(res_small_spont_edgeR, res_small_spont_DeSeq,
by.x="row.names", by.y="row")
merged_res_small_spont_minus_3<-
merged_res_small_spont_minus_3[merged_res_small_spont_minus_3$PValue<=0.01 &
merged_res_small_spont_minus_3$pvalue<=0.01,]
dim(merged_res_small_spont_minus_3) #165
head(merged_res_small_spont_minus_3)
tail(merged_res_small_spont_minus_3)
merged_res_small_spont_minus_3<-
merged_res_small_spont_minus_3[with(merged_res_small_spont_minus_3, order(PValue)), ]
```

Merge the results for initial and validation analysis

```
merged_res_small_spont_validated<-merge(merged_res_small_spont,
merged_res_small_spont_minus_3, by.x="row.names", by.y="row.names", all.x=TRUE,
all.y=FALSE)
```
